# Pathophysiology of X-Linked Adrenoleukodystrophy: Updates on Molecular Mechanisms

**DOI:** 10.26502/jbb.2642-91280151

**Published:** 2024-06-14

**Authors:** Parveen Parasar, Navtej Kaur, Jaspreet Singh

**Affiliations:** 1 Department of Neurology, Henry Ford Health, Detroit, MI 48202, USA; 2 Department of Physiology, Michigan State University, Lansing, MI 48824, USA

**Keywords:** Metabolic, X-ALD, Biomarkers, Etiology, miRNA

## Abstract

X-ALD, an inherited monogenic metabolic disorder affecting the CNS and adrenal white matter, is caused by mutations in ABCD1 gene leading to defective fatty acid oxidation in the peroxisomes. This results in accumulation of very long-chain fatty acids, VLCFA, into brain, spinal cord, and body fluids. A single ABCD1mutation does not clearly explain the severity and diverse clinical spectrum of X-ALD phenotypes which suggests that not only genetic but also other modifier genes, epigenetic factors, and environmental factors play a role and contribute to neuroinflammation, mitochondrial dysfunctions, oxidative stress, and metabolic defects seen in phenotypes of ALD. In this review we discuss genotype and phenotype correlation and clinical spectra of X-ALD, previous and recent modifier genetic factors of X-ALD, including novel role of microRNAs (miRNAs) in pathology and as biomarkers. We also discuss the mechanistic interplay of miRNAs and metabolic pathways and potential of targeting miRNAs for X-ALD.

## Introduction

X-linked adrenoleukodystrophy (X-ALD) is an inborn neurometabolic genetic disorder which results in progressive demyelination in central nervous system (CNS), axonopathy in the spinal cord, and adrenal insufficiency [1]. The disease is monogenic peroxisomal disorder in view of its etiology as mutations in the *ABCD1* gene which encodes a peroxisomal ATP-binding cassette transporter for very long chain fatty acids ≥ C22:0 (VLCFA) into the peroxisome for β-oxidation [1, 2]. The defective peroxisomal β-oxidation causes accumulation of VLCFAs in all tissues, primarily affecting myelin, axons, the adrenal cortex, and testis [1, 3]. The incidence of hemizygotes (all phenotypes) and heterozygote female carriers occurs in 1 out of 16,800 newborns. While phenotype/genotype correlation does not exist, clinically ALD presents various distinct phenotypes which are based on the age of onset, rate of progression, and the site of first pathologic signs [4, 5]. The nervous system pathology is variable and might be rapidly progressive, inflammatory cerebral demyelination (cerebral ALD) or a more slowly progressive axonopathy in the ascending and descending spinal cord tracts (adrenomyeloneuropathy, AMN). The most severe form is cerebral or juvenile ALD manifested by inflammatory demyelination of the CNS, adrenocortical atrophy, and a rapidly fatal clinical course resulting from the CNS lesion. AMN causes progressive dying-back axonopathy and mostly seen in males. As in many X-linked diseases, female carriers remain asymptomatic, however, many women develop AMN-like symptoms between 50 and 60 years.

### Phenotypes/Spectrum of X-Ald

X-ALD has diverse clinical presentation which can be asymptomatic or present in rapid progressive phenotypes. Male patients present three main forms: Cerebral demyelinating form, CALD; Spinal cord demyelination and axonal degeneration in AMN; and Adrenocortical insufficiency shown as Addison-like form.

### Phenotypes in male X-ALD patients

#### Cerebral Adrenoleukodystrophy (cALD)

cALD is the most rapidly progressive phenotype of X-ALD occurring in children (35–40%) aged 2–12 years and characterized by sudden inflammatory progressive intellectual, psychic, visual, and gait disturbances. Onset occurs with deficits in cognitive abilities such as visuomotor and visuospatial functions resulting in decline in school performance in boys and adolescents. Further progression occurs with more visible neurologic dysfunctions such as hyperactive behavior, apraxia, auditor impairment, astereognosis, hemiparesis or spastic tetraparesis, cerebellar ataxia, and seizures. Patients show extremely rapid progression and lose the ability to walk and comprehend the language. Within a few weeks they become blind, unable to speak, bedridden, and require full time assistance with feeding with nasogastric tube and within two to four years they succumb to death or remain in vegetative state for several years. Although extremely rare, spontaneous arrest of disease progression may occur in boys with inflammatory cALD [6, 7].

### Adolescent and adult cALD

Cerebral ALD occurs less frequently (20%) in adolescents (AdolcALD) (10–20 years) or adults (acALD) (>20 years). Symptoms in these patients strongly resembles in cALD, however, the initial progression of the symptoms is slower. Adolescents show clinical features of cerebral involvement. In adult cALD, symptoms like schizophrenia, spastic paraparesis, seizures, and dementia are seen [3–5].

Some adolescents or adults (~10%) with or without AMN may not develop rapid progressive form of cerebral neuroinflammatory stage of the disease. The cerebral demyelinating process arrests spontaneously, and patients remain stable for a decade or longer and may show sudden onset with rapid full progression of neurologic deterioration and inflammation [5].

### Adrenomyeloneuropathy (AMN)

AMN is gradually developing with core clinical feature as non-inflammatory axonopathy mainly affecting males aged 20–30 years and females between 40 and 50 years [3–5]. Early symptoms are progressive weakness in legs with impaired vibration sense in lower limbs, sphincter disturbances and impotence. AMN could show pure-AMN or AMN-cerebral variants where pure-AMN patients show gait disturbance and bladder dysfunctions due to pathology in spinal cord. On the other hand AMN-cerebral type manifest cerebral inflammation as well as pure-AMN features [8, 9]. The mechanisms of conversion of pure-AMN form into AMN-cerebral form is yet to be disclosed.

### Addison only phenotype

Rare cases show distinct Addison only or adrenocortical dysfunction without neurological involvement and are characterized by fatigue, hypotension, and bronzing of the skin. These patients show elevated plasma concentrations of adrenocorticotrophic hormone (ACTH) and lower concentration of cortisol. Majority of Addison-only patients develop cALD or AMN and develop neurological involvement [10, 11].

### Asymptomatic Males and Females

In 20% - 50% of heterozygotes or female carriers develop AMN-like symptoms with an average onset between 40 and 50 years [8, 10]. These symptoms may include disturbance in gait, dysuria, and urgency. Ameliorated symptoms in females are due to X-inactivation where enough ABCD1 functional activity is provided by the normal allele located on the other set of X chromosome in the cells. Although rare, but some females also develop cALD and adrenal insufficiency [12–14] which may result due to skewed X-inactivation and preferential expression of the X chromosome carrying the mutant ABCD1 allele [15–17]. The mechanisms contributing to skewing of the X-inactivation are not fully understood.

### Pathophysiology and Molecular Mechanisms Underlying in X-Ald Phenotypes

The definitive molecular basis and underlying mechanisms of clinical variants of X-ALD are yet to be understood. However, biochemical, lipidomic, genetic, and to some extent immunologic mechanisms have been reported which contribute to pathophysiology of X-ALD and its variants.

### Pathophysiology of cALD

**Figure 1:**
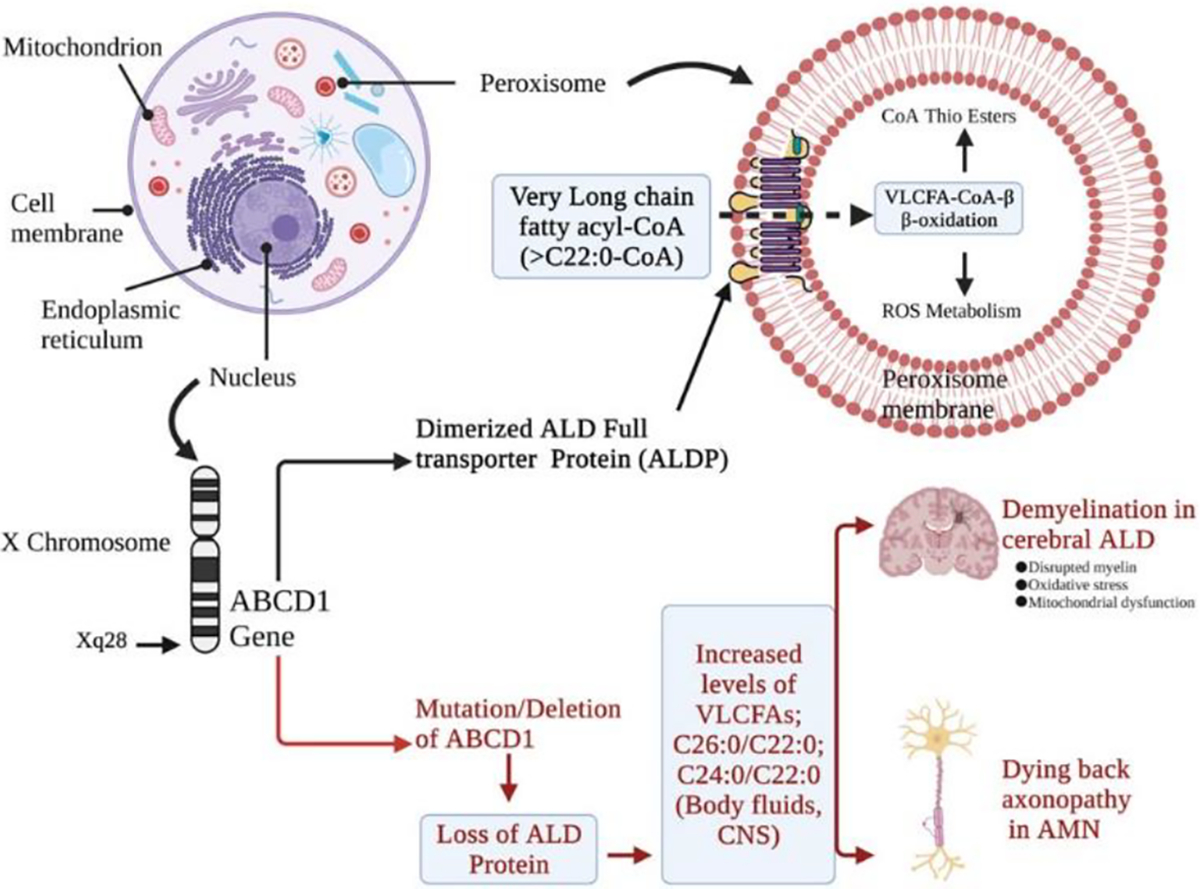
Molecular and biochemical features of X-ALD phenotypes

Pathologic hallmark of cALD is inflammatory cerebral demyelination (Figure 1). The extensive inflammatory demyelinating lesions favor the parieto-occipital regions of the cerebral white matter. The inflammatory demyelination, starting at the midline of corpus callosum symmetrically progresses outward in the hemispheres coinciding with neurologic decline resulting in vegetative state or death within 3–5 years. Studies exist which show that oxidative stress is the main contributor to cerebral inflammation and possibly AMN. Postmortem brains of X-ALD patients showed increased levels of heme-oxygenase-1 (HO-1) and manganese superoxide dismutase (MnSOD) in the [18].

Elevated levels of saturated, unbranched VLCFAs, particularly tetracosanoic (C24:0) and hexacosanoic acids (C26:0) in tissues and body fluids leads to biochemical alteration in all clinical variants of X-ALD. Normal degradation (known as β-oxidation) of VLCFAs requires a separate set of enzymes located within the peroxisomal matrix. In X-ALD, the activity of peroxisomal enzyme, very long-chain acyl-CoA synthetase, which degrades VLCFA into their CoA thioesters is reduced and thus β-oxidation is decreased leading to accumulation of VLCFAs in not only blood, tissues, and plasma [5, 8] but also complex lipids such as phosphatidylcholine [19], proteolipids [20], and ganglioside [21]. Theda et al. showed that in normal appearing postmortem brains of cALD patients, C26:0 level was elevated ~39 fold compared to that of controls and specifically localized in phosphatidylcholine fraction. Impaired degradation of VLCFAs and deposition of VLCFAs in complex lipids are directly involved in pathology of the cerebral inflammatory form of X-ALD. Peroxisomal β-oxidation is decreased to ~30% of normal in cultured human fibroblasts of X-ALD patients [22]. *In vitro* study performed in artificial phospholipid vesicles showed that accumulation of VLCFAs in within myelin could lead to destabilization of myelin sheath followed by demyelination [23]. Although the definitive mechanism is poorly understood, it is possible that the degree of demyelination varies based on the amount of VLCFAs in myelin sheath. cALD patients showed higher amount of VLCFAs in white matter compared to that of AMN patients. Induced pluripotent stem cells (iPSC)-derived oligodendrocytes from these patients also showed more accumulation of VLCFAs when compared to the AMN patients [24]. We reported a similar increased accumulation of VLCFA (C26:0) in cALD patient iPSC-derived astrocytes [25]. In other theories, autosomal genes [26–28] have been proposed to play a role in clinical variant of X-ALD. Genes or enzymes such as ELOVL6 and ELOVL1 (elongases) involved in endogenous pathways of VLCFA generation may play role in cerebral demyelination [29, 30].

### Pathophysiology of neuroinflammation

Blood-brain integrity is important factor in X-ALD. While in 10–15% X-ALD patients who develop cerebral demyelination spontaneous arrest of demyelinating process occurs with unaltered blood-brain barrier, majority of cALD patients show disrupted blood-brain barrier. Through the opened barrier invade the mononuclear cells predominantly macrophages mostly containing myelin degradation products [28]. Moreover, complex lipids such as lysophosphatidylcholine containing incorporated VLCFA might lead to activation and apoptosis of microglia [31] and thus leading to neuroinflammation and elevated proinflammatory cytokines in cerebrospinal fluid of cALD patients [32]. Invading macrophages and activated microglia cells accumulate VLCFA generated from phagocytosed myelin. Intracellular aggregates affect immune functions of macrophages and cytokine secretion and make these cells resistant to anti-inflammatory therapies. Furthermore, in some areas with disrupted blood-brain barrier, T cells (specially CD8) and to lesser extent, B cells infiltrate resulting in cytolysis of oligodendrocytes, however, this does not induce anti-CNS immunity [33, 34].

In compromised barrier and increased permeability, the VLCFA-containing proteolipid and ganglioside antigens are recognized and presented by CD1 molecules. Gangliosides, proteolipids, and phosphatidylcholine lipids containing VLCFA are presented by CD1 molecules to T cells and could thus trigger autoimmune responses in X-ALD brains. CD1 molecules present foreign and lipid-self antigens to T cells in major histocompatibility complex (MHC)-unrestricted manner and have been revealed most prominently in cALD lesions [35, 36]. Due to brain-specificity of some gangliosides it is highly likely that inflammatory reaction is mostly in X-ALD patient brains and not in other affected tissues such as peripheral nerves, adrenocortical cells or Leydig cells in testes. Similarly, proteolipid is abundant component of myelin containing VLCFA and therefore could trigger an autoimmune reaction after myelin disruption in cALD patients. Transplantation with hematopoietic stem cells or *ex vivo* correction of gene correction of autologous HSCs is efficient therapy for inflammatory form of X-ALD in early age with no cross correction of other cell-types after HSCT [37]. Mechanism of pathogenesis in childhood and adult cerebral disease show no difference in mechanisms of pathogenesis and reveal oxidative stress, infiltration of macrophages and activated/apoptotic microglia in the peri-lesion in affected brains.

### Pathophysiology of AMN

The main symptoms of AMN are adrenal insufficiency, gait difficulties, and bladder and bowel problems [9]. About 30% of AMN patients develop neuroinflammation and demyelination and progress to cALD and death [5, 8]. Patients develop adrenal insufficiency independent of myeloneuropathy with no correlation between severity/duration of endocrine dysfunction and myeloneuropathy. AMN patients do not show apparent neuronal loss or apoptosis as evidenced by histologic studies in their dorsal root ganglia; however, neuronal atrophy with inclusion body accumulation in mitochondria are seen [18]. As with cALD, oxidative stress also has been detected in both cALD and AMN patients. Both cALD and AMN patients showed decreased glutathione in lymphocytes compared to that of controls [38, 39]. Both AMN and cALD fibroblasts show increased oxidative stress when treated with VLCFA [40]. Application of C26:0, C24:0, and C22:0 and not C16:0 VLCFA leads to cytotoxicity in oligodendrocytes, astrocytes, and neurons in rats [41]. Excess C26:0 may cause ROS production in mitochondria which is detrimental to DNA and proteins and lead to altered and inefficient oxidative phosphorylation and axonopathy. Silencing of ABCD1 and ABCD2 in mouse primary astrocytes *in vitro* caused an increase in C_26:0_/C_22:0_ ratio as well as in the levels of C_26:0_ compared with control and scrambled RNA (ScrRNA)-treated astrocytes. ABCD1/ABCD2 silencing also induced expression of TNF-α, IL-1β, and inducible nitric oxide synthase (iNOS) in astrocytes suggesting that VLCFA accumulation is linked to inflammatory response in astrocytes [42].

Peroxisomes are important for maintaining axonal integrity and their impairment could lead to axonal degeneration. In mice with deficient peroxisomes in astrocytes and neurons, no axonal degeneration has been detected [43], however, a selective peroxisomal deficiency in oligodendrocytes leads to early axonal loss and neuroinflammation with no demyelination [44] suggesting that oligodendrocyte-axonal and myelin-axonal integrity is important in AMN. A disturbance in any of these interactions may lead to mitochondrial dysfunction and axonopathy [8].

### Metabolic defects in X-ALD/Biochemical abnormality

#### Biochemical changes in brain (cALD)

In addition to ABCD1, ABCD2 and ABCD3 are also localized in the peroxisomes [45–47]. Due to homology of ABCD1 with ABCD3 and ABCD2, there has not been complete abolition of β-oxidation of VLCFA in cells and tissues and primary fibroblasts of X-ALD patients [48]. However, ABCD3 does not compensate fully with the deleted ABCD1 as its efficiency is 45 times lesser than ABCD1. ABCD2 is the closest homolog of ABCD1, but it is expressed inadequately in normal conditions and thus cannot account for the residual β-oxidation in fibroblasts. Varying expression levels of these proteins in different cell types produce differing extents of metabolic defects in X-ALD patients due to the fact that all three isoforms can contribute to the transport and degradation of VLCFA. The accumulated VLCFA are synthesized endogenously upon elongation of long- and very long-chain fatty acids [49]. VLCFA are intracytoplasmic lamellae and lamellar lipid inclusions in brain macrophages as well as adrenal cells, Schwann cells, and were also detectable in fibroblasts, blood, and plasma of X-ALD patients [50]. Normal myelin contains mostly long-chain fatty acids (C16 to C20) in contrast to X-ALD brain which contains large amounts of VLCFA which further induces apoptosis of oligodendrocytes and activation of microglia and cytokine secretion. High amounts of VLCFA causes structural damage in the membrane bilayer and thus CNS functions are altered. This suggests that accumulation of VLCFA in the white matter may result in inflammation and demyelination.

### Biochemical changes in spinal cord (AMN)

Due to VLCFA accumulation, the axon-myelin interaction is disrupted and axonal support by oligodendrocyte/Schwann cells is lost which lead to mitochondrial dysfunction and oxidative stress. The dorsal and lateral spinal cord columns show axonopathy and lateral column shows atrophy. Many mitochondria in AMN neurons at the ultrastructural level show lipid inclusions. Neuronal atrophy along with the decreased number of large neurons also is seen [51].

### Biochemical changes in X-ALD Adrenal cortex

In the adrenal gland, ABCD1 is strongly expressed in the cortex but not in the medulla, on the other hand, ABCD2 shows stronger expression in the medulla and not in the cortex. It is reported that the adrenal pathology in X-ALD is limited to the cortex only and its degeneration leads to Addison’s disease in X-ALD patients.

### Oxidative stress/Endoplasmic stress/Inflammation in X-ALD

VLCFA accumulation disrupts the integrity of the plasma membrane while interdigitating through the lipid bilayer. The accumulated VLCFA such as hexacosanoic acid (C26:0) directly increases steady-state ROS production depleting GSH and decreasing mitochondrial membrane potential. It further causes endoplasmic reticulum (ER) stress, mitochondrial dysfunction, and oxidative stress and resulting in apoptosis and initiating and favoring the demyelination process. Not only unsaturated but also monounsaturated VLCFA (C26:1) fatty acid also generate ROS in X-ALD fibroblasts and generate free radicals which result in lipid peroxidation whose byproducts cause severe damage to the cells [40].

A study revealed that master regulator of the endogenous antioxidant response nuclear factor 2 erythroid 2-like 2 (NRF2) in both ABCD1-KO mice and X-ALD human fibroblasts has impaired activity due to impaired AKT/GSK-3β-NRF2 axis. Defective phosphorylation of AKT resulted in the activation of GSK-3β which further represses NRF2 [52]. In a recent study, abnormal levels of cannabinoid receptor 2 (CB2r) and related endocannabinoid enzymes were found in the brain and peripheral blood mononuclear cells (PBMCs) of X-ALD patients and in the spinal cord of a murine model of X-ALD. They also reported that the use of selective agonist of CB2r improved metabolic disturbances by rescuing GSK-3β-NRF2 axis and improved oxidative stress by induction of NRF2 antioxidant pathway [53]. Trauma or injury to the head triggers or activate symptoms in otherwise asymptomatic or arrested X-ALD patients [54]. It is possible that head trauma causes inflammatory response and thus followed by mitochondrial dysfunction, oxidative stress, and disruption of the BBB initiating the cerebral inflammatory demyelination and appearance of symptoms [55]. Oxidative modifications were seen not only postmortem brains of X-ALD patients but also skin-derived fibroblasts, plasma, and blood cells. Nitric oxide synthase 2 (NOS2) and lipid peroxidation products were increased in the inflammatory lesions and plasma of ALD with astrocytosis and microgliosis [18, 56, 57].

### Significance of inflammation and oxidative stress in X-ALD

Accumulation of VLCFA in lymphoblasts induces release of proinflammatory cytokines as well as ROS. Inflammation and oxidative stress are tightly intertwined and regulated by redox sensors which are regulators of inflammatory responses. One such sensor is nuclear factor kappa B (NF-κB) and the other is NRF2. NF-κB has redox-reactive cysteine resides which are modifiable by ROS. Nitrosylation of cysteine residues suppresses the translocation and DNA binding of NF-κB while H_2_O_2_ activates these parameters when interact with cytokines [58, 59]. NRF2 is a transcription factor that coordinates the synthesis of antioxidant systems and reducing systems via antioxidant response elements (ARE) located in the target genes. Oxidative stress modifies thiol groups in Keap1 gene activating NRF2 and thereby releasing this transcription factor. NRF2 lacking neurons are more susceptible to lipotoxic and excitotoxic damage and thus suggest the important role of this factor in oxidative stress [60]. Free radicals caused by oligodendrocytes, or the glial cells may initially induce protective inflammatory response via NF-κB; however, further accumulation of VLCFA and an impaired NRF2 mediated protective mechanism could aggravate glial inflammation and aberrant activation in cALD.

### Modifier genes in X-ALD

Mutation in ABCD1 gene alone is not adequate to define clinical variants of X-ALD observed in patients. Phenotypic variability in X-ALD has been studied in various studies and identical defects in the ABCD1 gene have been reported in different variants on X-ALD [61, 62]. Relative involvement of modifier genes/factors such as genetics, epigenetic, and environmental modifiers may change severity and progression of a disease and thus phenotypic variation in X-ALD does not exactly depend on the genetic mutation in ABCD1. Combination of these modifiers may vary among individuals and therefore may result in different phenotypes despite the single gene mutation, however, identifying association of these modifiers with gene involved in the disease is highly challenging. Studies exist which have investigated the roles of various modifier genes involved in VLCFA metabolism, inflammatory pathways, methionine metabolism, and bile acid metabolism.

### Genes associated with VLCFA metabolism.

In the peroxisomal matrix, saturated and unbranched VLCFA undergo β-oxidation pathway and are metabolized [63]. In X-ALD, the defective ATP-binding cassette (ABC) transporter protein, adrenoleukodystrophy protein (ALDP) causes accumulation of VLCFA, particularly C26:0 in various tissues and are incorporated into different complex lipids [8]. In addition to ABCD1 which encodes ALDP, ABC-transporters include ALDRP, and PMP70 encoded by ABCD2 and ABCD3 genes. ABCD2-gene does not contribute to susceptibility for cerebral demyelination owing to its similar concentrations in unaffected brain white matter in different X-ALD phenotypes [64]. Contrary to that ABCD4 gene expression is associated with brain demyelination in cALD, pure-AMN, and AMN-cerebral forms. Other modifier gene which result in accumulation of VLCFA in cells and tissues is ELOVL1 which is responsible for excessive lengthening of long chain fatty acids to VLCFA. Knock down of *ELOVL*1 has resulted in reduction of C26:0 concentration in X-ALD fibroblasts; however, its expression is unchanged in X-ALD fibroblasts [30]. During their metabolism, VLCFA converts to their coenzyme A derivatives upon actions of acyl-coA synthetases. BG1 gene which encodes non-peroxisomal synthetase which activates VLCFA to its coenzyme A derivatives has been correlated with cerebral demyelination [64]. Alternative route of VLCFA metabolism is through ω-oxidation which uses cytochrome P450 system to convert VLCFA into dicarboxylic acids. Polymorphism of CYP4F2 gene that encodes a key enzyme in ω-oxidation is associated with the increased chances of acquiring cALD in male Caucasians [65].

### Role of AMP-activated protein kinase (AMPK) in X-ALD

Our group documented the novel loss of metabolic gene, AMP-activated protein kinaseα1 (AMPKα1) in cALD patient-derived fibroblasts and lymphocytes [66], postmortem brain tissue [67] and in X-ALD patient iPSC-derived astrocytes [25]. Furthermore, we demonstrated that knockdown of AMPKα1 in ABCD1-KnockOut (KO) mice induced proinflammatory cytokine response and mitochondrial dysfunction [68] which suggests that loss of metabolic gene AMPKα1 contributes to development of inflammation [69–72]. We demonstrated that AMPK activator, Metformin, induced AMPKα1 in X-ALD patient-derived fibroblasts in a dose-dependent manner. Metformin treatment also decreased VLCFA levels and proinflammatory cytokine expression in X-ALD patient-derived cells and increased ABCD2 levels in X-ALD patient-derived fibroblasts and ABCD1-KO mice primary mixed glial cells. *In vivo* treatment with Metformin induced mitochondrial oxidative phosphorylation protein levels in the brain and spinal cord of ABCD1-KO mice [67]. This shows that AMPKα1 can be a strong potential modifier gene in X-ALD. Alterations in metabolic regulation are important in diseases, cancer, and inflammatory conditions. In limited oxygen or hypoxic condition, mitochondrial oxidative metabolism is restricted with conversion of pyruvate into lactate. Despite plentiful oxygen, tumor cells show lactate deposition also known as Warburg effect [73]. Similarly, activated immune cells such as monocytes and macrophages depend on glycolysis as source of metabolic energy [74, 75]. In activated lymphocytes and during phagocytosis glycolysis and glutamine metabolism are increased as shown in mice and rats [76, 77]. Among other metabolic genetic factors, methionine metabolism pathways such as cystathionine β-synthase (CBS), methionine synthase (MTR), methylenetetrahydrofolate reductase (MTHFR), and dihydrofolate reductase (DHFR) have been studied [78–80].

### Genes related to inflammation

Inflammatory response to accumulated VLCFA occurring in the brain and spinal cord can be due to underlying genetic factors such as human leukocyte antigen (HLA) class II antigen, DRB1. Allele HLA-DRB1*16 plays a role in synthesis of peptide receptors playing a central role in antigen presentation and immune responses. Berger et al. disclosed a significant association between X-ALD and HLA-DRB1*16 allele; however, this association was not dependent on cALD [81]. A later study with larger sample size did not show association between DRB1*16 and X-ALD [82]. Among other key genes of inflammation are tumor necrosis factor-α (TNF-α) and interleukin 6 (*IL6)* were also investigated and no interactive association of these genes with any phenotype was elucidated [82, 83].

### Epigenetic modifiers of X-ALD

Modifiers of epigenetic nature are DNA methylation, post-translational modifications of histones (methylation, phosphorylation, and acetylation), and post-transcriptional regulation by non-coding RNA. Schlüter et al. showed in their genome-wide DNA methylation study of unaffected frontal brain white matter of cALD and AMN patients that oligodendrocyte-differentiation related genes such as MBP, CNP, MOG, and PMP1 were hypermethylated and the genes of immune functions such as IFITM1 and CD59 were hypomethylated [84]. MicroRNAs (miRNAs) are small non-coding RNAs that regulate post-transcriptional regulation of gene expression. Single miRNA can target multiple mRNAs, as such each miRNA can regulate multiple metabolic pathways. We reported the first role of miRNA in regulating the inflammatory response in cALD patient lymphocytes [85]. In one study, decreased expression of miR-196a and increased ELOVL, IKKα, IKKβ, MAP4K2, and MAP3K2 in cerebral ALD when compared to that in AMN and control fibroblasts implying the role of miR196-A in regulation of inflammatory signaling pathway in cALD brain [86].These studies profiled limited set of miRNAs. To identify global changes in miRNA relevant to neuropathology in X-ALD, we recently performed small RNA sequencing (MiSeq) of normal looking and lesion areas of cALD postmortem human brain [87]. The study identified several known and novel miRNAs in postmortem brain white matter from cALD patients [87]. Atleast three miRNA were identified (miR-2114, miR-3063, miR-6131) that were previously unreported in any neurodegenerative disorder [87].

X-ALD is a disorder of inborn error of metabolism and metabolic disturbances are expected. We reported the loss of metabolic enzyme AMP-activated protein kinase (AMPKα1) in fibroblasts, lymphocytes and postmortem brain tissue and in iPSC-derived astrocytes of patients with severe cALD phenotype [25, 66–68]. Mouse model of X-ALD (Abcd1-KO) does not develop neuroinflammation characteristic of human cALD [88–90]. Mixed glial cells from Abcd1-KO mice, deleted for AMPKα1 produced a neuroinflammatory response and mitochondrial dysfunction (i.e., decreased mitochondrial oxygen consumption rate)[68] and a similar decrease in mitochondrial oxygen consumption rate was seen in cALD patient iPSC-derived astrocytes [25]. Global untargeted metabolomics is powerful tool that can capture changes in large number of metabolites and identify multiple dysregulated biological networks. By global untargeted profiling of metabolites in human postmortem cALD brain we identified several metabolic pathways relevant to X-ALD pathology [87]. Employing a novel strategy we combined the data from small RNA sequencing (MiSeq) and untargeted metabolomics in cALD postmortem brain tissue we identified novel interaction between miRNA regulation of neurodegenerative pathways, mitochondrial energy metabolism and lipid and carbohydrate metabolism [87]. This provides a novel line of investigation on role of miRNAs as upstream regulators of neuroinflmmatory and demyelination pathways in cALD.

### Other X-ALD modifiers

**Figure 2:**
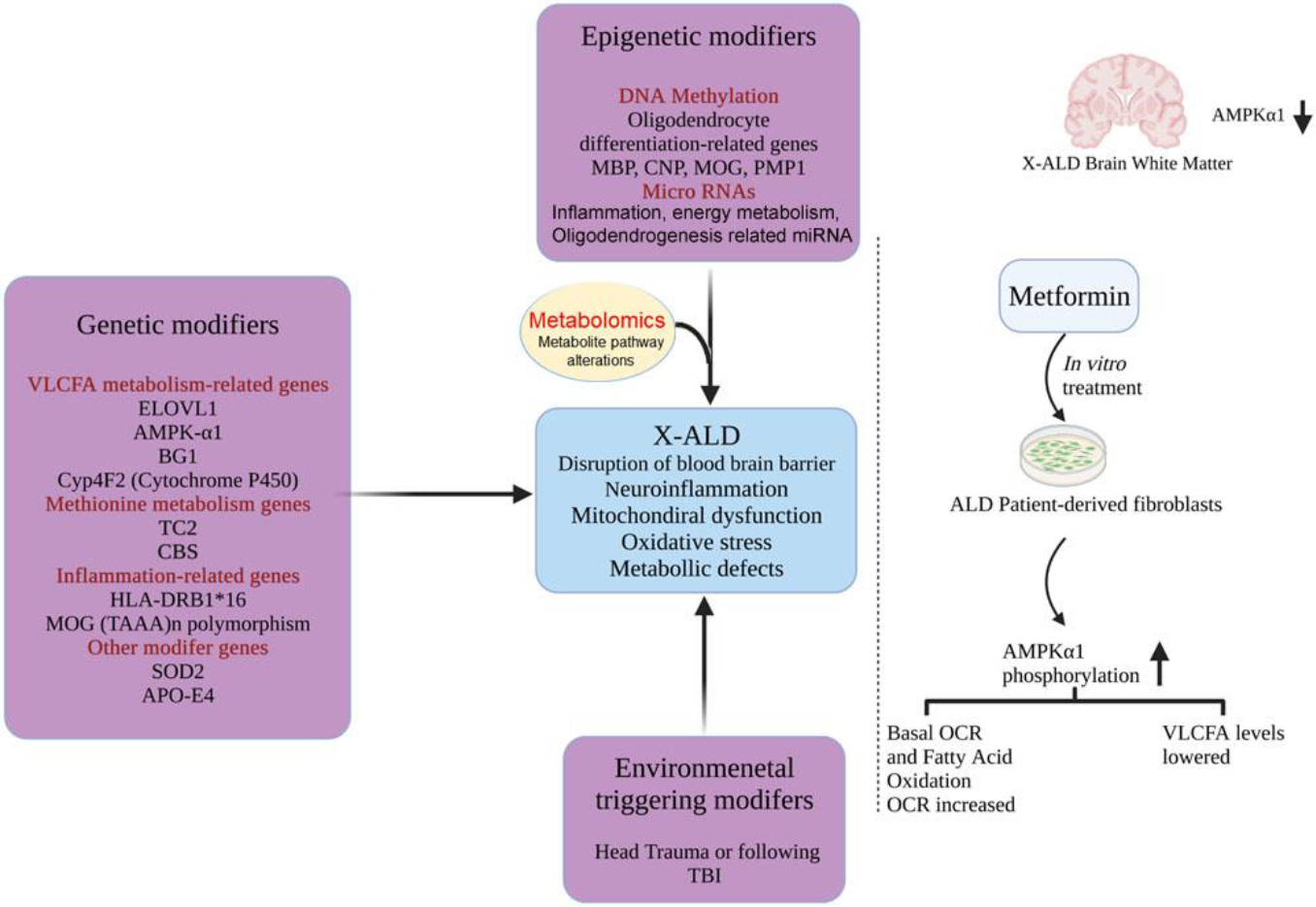
Role of modifiers in development of X-ALD phenotypes.

Dying-back axonal degeneration in the spinal cord is the characteristic of AMN form of X-ALD. Reactive oxygen species (ROS) are unstable oxygen-containing molecules which react with other molecules in a cell. A buildup of ROS in cells may damage RNA, DNA, and proteins inside cells as well as result in inefficient oxidative phosphorylation which further cause axonopathy. Enzyme, mitochondrial superoxide dismutase (SOD2), detoxifies ROS and its reduced activity has been associated with adolescent cerebral, adult cerebral X-ALD, and AMN cerebral patients [91]. Apolipoprotein E (APOE), a protein responsible for transport of lipoprotein-mediated lipid transport across organs, has been associated with severity of cerebral diseases in cerebral ALD. APOE has three isoforms, APOE2, APOE3, and APOE4. APOE 3, by downregulating cyclophilin A (CypA), a proinflammatory protein thus maintaining blood brain barrier (BBB) integrity. Male X-ALD patients harboring APOE4 haplotype in this study showed greater cerebral involvement [92]. A list of modifiers is depicted in the figure 2.

### Prognostic and diagnostic biomarkers of X-ALD

β-oxidation occurs in the peroxisomal matrix under which acyl-CoA enzymes degrade saturated, unbranched VLCFA (fatty acyl chain length >C22). In X-ALD patients, saturated VLCFA, especially C26:0, accumulate in tissues and body fluids and elevated plasma VLCFA is still the best initial biomarker for X-ALD. VLCFA-containing lysophosphatidylcholine has been demonstrated to induce microglia apoptosis and macrophage recruitment from the periphery. The metabolite, 1-hexacosanoyl-2-lyso-sn-3-glycero-phosphorylcholine (26:0-lyso-PC) is a diagnostic marker for X-ALD and also a candidate for prenatal analysis and neonatal screening for X-ALD at the Kennedy Krieger Laboratory and Mayo Clinic [93]. In another study, C26:0-Carnitine has been shown to be elevated in brain and spinal cord from ABCD1-KO mice, and bloodspots from mice and ALD patients. Furthermore, in our recent study, we performed untargeted metabolomics and miRNAseq on mild and sever AMN and age and sex-matched healthy human plasma and observed a significant clinical correlation for 7-alpha-hydroxy-3-oxo-4-cholestenoate (7-HOCA), dehydroepiandrosterone sulfate (DHEA-S), hypoxanthine, and miRNA-432–5p [94]. In this study, we documented the first evidence that metabolites and miRNAs can be clustered by disease severity groups and thus provide as a tool to identify potential biomarkers of clinical disease severity in AMN patients.

### Therapies in X-ALD

Presently, there is no satisfactory treatment for X-ALD. The most severe ALD phenotype cerebral childhood ALD is rapidly progressive inflammatory brain demyelination with a fatal outcome unless diagnosed early and treated with hematopoietic bone marrow or cord blood transplantation [95, 96]. A matched related than an unrelated donor gives more improved results [97]. The mechanism of HSCT-mediated arrest of the brain inflammation in X-ALD is unclear; however, the success of the HSC gene therapy shows that only a partial correction of HSC progeny is necessary to halt cerebral disease [37, 95]. The most promising result so far are from *ex vivo* gene therapy trial to stop cALD progression by using autologous HSCT with *ex vivo* gene transfer to increase ABCD2 expression using a Lentiviral vector [96]. Our laboratory has used Metformin, an anti-diabetic drug for type 2 diabetes to test its therapeutic potential for X-ALD. We demonstrated that metformin induced peroxisomal ABCD2 *in vitro* and *in vivo*. Metformin at 100mg/Kg *in vivo* in drinking water induced ABCD2 levels and mitochondrial oxidative phosphorylation in the brain and spinal cord of ABCD1-KO mice [67]. In our study, metformin acted via APMK-dependent mechanism and we found that it increased partial and total AMPKα1 in AMN and cALD patient-derived cells consistent with a previous report [98] suggesting metformin can be a potential therapeutic agent for X-ALD. In our recent study, we demonstrated that PXL-065, a stabilized form of R-pioglitazone (a marketed antidiabetic thiazolidinedione (TZD) drug, suppresses elevated VLCFA levels (C26:0 and C26:0/C22:0) in AMN patient-derived fibroblasts. Furthermore, PXL065 improved mitochondrial functions by inducing overexpression of ABCD2 and ABCD3 transporters [99]. In another study, we harnessed the potential of an AMPKα1 activator, PXL770 and demonstrated its potential to normalize VLCFA levels in cALD and AMN fibroblasts and lymphocytes after a week of treatment. PXL770 also improved mitochondrial functions in cALD and AMN fibroblasts by upregulating ABCD2 expression. Moreover, we observed improved neuroinflammatory gene expression markers, NOS2, NF-κB, CCL5, and CCR3 in cALD lymphocytes [100]. These results indicate that PXl770 can have beneficial effects to ameliorate the proinflammatory phenotype of the diseased cells.

No definitive therapy has been yet identified to halt the axonopathy in AMN. The combination of antioxidants, α-tocopherol (vit E), N-acetylcysteine (NAC), and α-lipoic acid (LA), has been shown to be synergistic *in vitro*, halting the clinical progression and axonal damage in a murine model of AMN [101]. Adrenal insufficiency is monitored closely and is treatable with hormonal supplementation, neurological aspects of X-ALD are far more challenging to treat. With the rising rate of newly diagnosed ALD-newborns, there is an urgent need for novel therapies. All families with male and female ALD infants should receive genetic counseling. Male infants should be monitored by an endocrinologist for the adrenal insufficiency follow-up. They should also receive annual magnetic resonance imaging (MRI)between 1 to 2 years of age and then every 6 months until 12 years of age and yearly thereafter to monitor any early cALD signs and if needed treated with an HLA-matched donor or *ex vivo* gene transplant [102].

## Conclusions

X-ALD is a metabolic monogenetic disorder which presents clinically varying phenotypic spectra which do not correlate with their genotypes. Aberrantly expressed miRNAs affect disease pathology in AD, PD, MS, HD and ALS. Emerging data from patient plasma samples and postmortem brain white matter of cALD patients [87, 94] documented by our laboratory identified several overlapping and novel miRNA related to neuroinflammation, oligodendrocyte pathology and metabolic alterations consistent with X-ALD pathophysiology. Downregulation of AMPK and dysregulation of metabolic pathways in cALD and the emerging role of miRNA may provide clues to upstream regulators of neuroinflammation in cALD. Majority of central nervous system neuroinflammatory and demyelinating diseases, including X-ALD, lack effective treatment. miRNA-based therapeutics have entered clinical trials for cancer and infectious diseases. Consistency of miRNA expression within the biological replicates of plasma in AMN and cALD phenotypes and postmortem brain tissue in cALD highlights their remarkable potential value for diagnosis, prevention, prognosis, and therapeutic value in X-ALD.
